# Translatability of preclinical to early clinical tolerable and pharmacologically active dose ranges for central nervous system active drugs

**DOI:** 10.1038/s41398-023-02353-1

**Published:** 2023-03-01

**Authors:** Guilherme S. Ferreira, Francis M. Dijkstra, Désirée H. Veening-Griffioen, Wouter P. C. Boon, Huub Schellekens, Ellen H. M. Moors, Peter J. K. van Meer, Frederik E. Stuurman, Joop M. A. van Gerven

**Affiliations:** 1 Department of Pharmaceutics, Utrecht Institute for Pharmaceutical Sciences, Utrecht University, Utrecht, The Netherlands; 2 Centre for Human Drug Research, Leiden, The Netherlands; 3 Leiden University Medical Centre, Leiden, The Netherlands; 4 Copernicus Institute of Sustainable Development, Innovation Studies, Utrecht University, Utrecht, The Netherlands; 5 Medicines Evaluation Board (CBG), Utrecht, The Netherlands; 6 Central Committee on Research Involving Human Subjects (CCMO), Leiden, The Netherlands

**Keywords:** Scientific community, Clinical pharmacology

## Abstract

The primary purpose of this study was to assess the translatability of preclinical to early clinical tolerable and pharmacologically active dose ranges for central nervous system (CNS) active drugs. As a part of this, IBs were reviewed on reporting quality. Investigator’s Brochures (IBs) of studies performed at the Centre for Human Drug Research (CHDR) reporting statistically significant results of CNS activity related to the drug’s mechanism of action were included. The quality of IBs was assessed based on the presence of a rationale for the chosen animal model, completeness of pharmacokinetic (PK) results in reporting and internal validity information of the preclinical evidence. The IB-derisk tool was used to generate preclinical and early clinical data overviews data. For each compound, the overlap between pharmacologically active dose ranges and well-tolerated levels was calculated for three pharmacokinetic (PK) parameters: human equivalent dose (HED), maximum plasma concentration (*C*_max_) and area under the curve (AUC). Twenty-five IBs were included. In general, the quality of reporting in IBs was assessed as poor. About a third of studies did not explore the entire concentration-effect curve (pre)clinically. Single dose tolerability ranges were most accurately predicted by *C*_max_. Human equivalent dose and AUC were the best predictors of pharmacologically active ranges. Tolerable and pharmacologically active dose ranges in healthy volunteers can be reasonably well predicted from preclinical data with the IB-derisk tool. The translatability of preclinical studies can be improved by applying a higher reporting standard in IBs including comparable PK measurements across all preclinical and clinical studies.

## Introduction

Drug development programs for neurological and psychiatric diseases have a high failure rate in both phase II and phase III [1, 2]. Reasons include the lack of safety and efficacy in clinical stages of drug development [1–4]. Next to that, a relatively large proportion of dose reductions of novel central nervous system (CNS) drugs is needed after marketing approval due to safety concerns [5]. Lastly, the poor translatability of preclinical experiments to clinical studies is often cited as a cause for these high attrition rates [6, 7].

For clinical researchers, the primary source of preclinical data for novel investigational products is the Investigator’s Brochure (IB). The IB is an obligatory part of a research file for clinical studies with an investigational medicinal product (IMP), which contains all nonclinical data relevant to studies in human subjects (supplemented with subsequent clinical results) [8]. According to the International Conference on Harmonization (ICH) guideline for Good Clinical Practice (GCP), the purpose of the IB is “to provide the investigators and others involved in the trial with the information to facilitate their understanding of the rationale for, and their compliance with, many key features of the protocol, such as the dose, dose frequency/interval, methods of administration and safety monitoring procedures” [8]. Minimum requirements for the IB are described in this guideline [8]. Despite this guidance, the content of IBs is highly variable in practice [9].

The European Medicines Agency (EMA) “guideline on strategies to identify and mitigate risks for first-in-human (FIH) and early clinical trials with investigational medicinal products” provides guidance on the quality and choices of preclinical safety and efficacy studies that should be performed prior to an FIH study and on how a safe starting dose should be determined [10]. According to this guideline, the starting dose should be based on both preclinical safety studies and efficacy or pharmacodynamic (PD) experiments [10]. Safety can be quantified by the no observed adverse effect level (NOAEL) in the most sensitive relevant species, and efficacy or (pharmacological) activity by estimations of the minimal anticipated biological effect level (MABEL), the pharmacologically active dose (PAD) and/or anticipated therapeutic dose (ATD) in humans [10]. In practice, however [10], the starting dose is often primarily based on safety considerations, as a fraction of the NOAEL [11]. This focus on NOAEL instead of a joint account of pharmacologically active levels can have disastrous consequences as observed with TGN1412 and BIA-10-2474 [12, 13] When TGN1412, a CD28 superagonistic antibody in development for the treatment of chronic lymphatic leukaemia and rheumatioid arthritis, was first administered to humans, it caused a cytokine release requiring intensive care treatment in all healthy individuals administered with the drug [12]. The starting dose for this study was based on the NOAEL and a factor 500 lower than the NOAEL, but at this dose cytokine release was already observed preclinically, indicating that the starting dose should have been even lower [12]. In the clinical trial with BIA-10-2474, a fatty acid amide hydrolase (FAAH)-inhibitor in development for diseases in which elevated endocannabinoid tone might be beneficial such as pain, glaucoma and post-traumatic stress disorder, several subjects developed neurological damage and one subject died [13]. The preclinical data identified safety concerns in the form of serious irreversible adverse effects that were observed at varying dose levels across species [14]. There were no pharmacological measures applied in the clinical study to counter this risk and dose escalation was based solely on tolerability findings [14]. Doses were escalated to *C*_max_ values ~12 times higher than levels of maximal FAAH-inhibition, leading to fatal consequences [14]. Not taking into account pharmacological active levels in early-phase clinical studies can also have less dramatic effects, as illustrated by the example of CEP-26401 [15]. The first clinical study of CEP-26401, a histamine-3 receptor antagonist developed to improve cognitive functioning in for example Alzheimer’s disease, schizophrenia or attention deficit hyperactivity disorder (ADHD), demonstrated improved cognitive functioning at the lowest dose tested [15]. Therefore, a second study was required to find the dose with the best balance between wanted (improved cognition) and unwanted (sleep inhibition) effects [15].

The aim of the current paper was to investigate the accuracy with which the IB preclinical package can predict tolerable and pharmacologically active dose ranges for CNS drugs in humans. We assessed the overlap between preclinical and clinical well-tolerated dose levels and pharmacologically active dose ranges. Furthermore, we checked whether both preclinical safety and in vivo pharmacology experiments were used when determining the starting dose for FIH studies as recommended by current EMA guidelines [10]. Also, we investigated the reporting quality in the IBs, and, for compounds where phase II or III clinical trials were performed in patients, we conducted an exploratory analysis of the translatability of preclinical pharmacological active ranges to therapeutic effective ranges.

## Methods

### Study and IB selection

We performed a structural review of all IBs in CNS drug development conducted between 2003 and 2019 at the Centre for Human Drug Research (CHDR). To adhere to confidentiality agreements agreed upon with sponsors and clients of the performed clinical trials, compounds were anonymised and no individual study results are described. In order to be suitable for analysis of the overlap of tolerable and pharmacologically active dose ranges, a dose range of tolerability and pharmacological activity had to be reported both preclinically and clinically. This meant that IBs were included if they included a statistically significant effect (as reported in the document) on any CNS activity related to the compound’s mechanism of action for at least two dose levels across the reported preclinical studies and in the associated Clinical Study Report (CSR). Preclinical in vitro studies were not taken into account as we aimed to investigate the predictivity of in vivo pharmacology experiments in animals. Studies testing combinations of drugs and studies performed for the purpose of method development with drugs already approved by the Food and Drug Administration (FDA) or the European Medicines Agency (EMA) were excluded. For approved drugs, the Summary of Product Characteristics (SmPC) is provided to the investigator instead of an IB and animal pharmacokinetic (PK) and PD data are not systematically reported. In case phase II and III therapeutic efficacy studies were performed for a compound, and an exploratory translatability assessment of therapeutic efficacy was performed.

### IB-derisk tool

In this paper, the IB-derisk tool was applied to IBs of included compounds. The IB-derisk tool (https://www.ib-derisk.org) is a tool that can be used to integrate preclinical and clinical data reported in the IB and for the comparison of preclinical studies results with predicted or emerging human data while the clinical study is ongoing [16]. An illustration of this can be found in the publication by Cohen and colleagues, who applied the IB-derisk tool to the BIA-10-2474 study [14].

For detailed information on the IB-derisk tool, we refer to the original publication [16]. In short, all reported in vitro findings and in vivo efficacy and safety findings and their according exposure parameters, such as the human equivalent dose (HED), maximum plasma concentration (*C*_max_) and total exposure (AUC) are entered in a spreadsheet-like document. By doing so, missing PK parameters can be estimated by interpolation and extrapolation, taking dose duration (single or multiple doses), route and bioavailability, species and sex into account. All effects are then colour-coded. Desired pharmacological effects are indicated by green, mild manageable adverse effects in yellow, more severe adverse effects that could not be accepted in a clinical situation but without unacceptable health risks by orange, and severe irreversible adverse effects in red. The no observed adverse effect level (NOAEL) in the most sensitive species is indicated in purple, and in vitro experiments are in light blue. By applying this colour coding, sorting the data on dose (HED), concentration or AUC provides a quick overview of dose-, concentration- or exposure-response patterns of pharmacological and toxicological effects, and of deviations from predictable relationships [16].

### Data collection

Data were collected from the source documents by the authors GSF and FMD. All in vivo behavioural pharmacology and safety preclinical study results reported in the included IBs were extracted, regardless of species or outcome. GSF and FMD also assessed the quality of reporting in IBs in a similar manner as described by Wieschowski et al [17]. As a part of this it was assessed whether a rationale for the chosen animal model was provided, the completeness of reporting of pharmacokinetic results of in vivo pharmacology experiments (including strain, sex, and route of administration) was assessed and internal validity information (randomization, blinding) of the preclinical evidence was assessed. GSF and FMD discussed their quality assessment in case there were any unclarities and the consensus answer was incorporated in Table 1.

**Table 1:** General characteristics of included IBs.

IB	Mechanism of Action	FIH	Starting dose rationale	Completeness of reporting; PK results of in vivo pharmacology experiments reported	Animal model justification	Internal validity
1	Cannabinoid receptor antagonist	No	In vivo pharmacology and safety	Dog: yes; Mouse: no; Rat: partial	None	No
2	Cannabinoid receptor agonist	Yes	Safety	Dog: partial; Micro-pig: no; Mini-pig: no; Mouse: no; Rat: partial	None	No
3	Orexin receptor antagonist	Yes	In vivo pharmacology and safety	Dog: partial; Guinea pig: no; Rats: partial	None	No
4	Cannabinoid receptor agonist	Yes	In vivo pharmacology and safety	Dogs: partial; Mini-pig: no; Mouse: no; Rat: partial	None	No
5	Cannabinoid receptor antagonist	Yes	In vivo pharmacology and safety	Dog: partial; Guinea-pig: no; Mouse: no; Rabbit: yes; Rat: partial	Pharmacological	No
6	Dopamine antagonist	No	Safety	Dogs: partial; Ferret: no; Guinea pig: partial; Mini-pig: yes; Monkey: yes; Mouse: partial; Rat: partial	Pharmacological	No
7	GABA receptor modulator	No	In vivo pharmacology and safety	Dog: yes; Guinea-pig: partial; Monkey: no; Mouse: no; Rat: partial	Pharmacological	No
8	Alpha 7 nicotinic acetylcholine receptor partial agonist	No	In vivo pharmacology and safety	Dog: partial; Monkey: partial; Mouse: partial; Rabbit: yes; Rat: partial	None	No
9	GABA-receptor partial agonist	Yes	Safety	Dog: partial; Mouse: no; Rat: partial	Commonly used/ pharmacological	No
10	Neublastin (GFRα3) co-receptor selective ligand	No	In vivo pharmacology and safety	Monkey: partial; Mouse: no; Rat: partial	Phenotypic	No
11	Orexin antagonist	Yes	Safety	Dog; partial; Guinea pig: no; Rat: partial	None	No
12	Histamine receptor agonist	Yes	Safety	Cat: partial; Dog: partial; Monkey: yes; Mouse: partial; Rat: partial	None	No
13	Histamine receptor antagonist	No	In vivo pharmacology and safety	Dog: yes; Monkey: partial; Mouse: no; Rat: partial	None	No
14	GABA-receptor selective positive allosteric modulator	Yes	In vivo pharmacology and safety	Dog: partial; Rat: partial, Mouse: partial	None	No
15	Alpha 7 nicotinic acetylcholine receptor agonist	No	In vivo pharmacology and safety	Dog: partial; Mouse: partial; Rat: partial	None	No
16	Orexin antagonist	No	In vivo pharmacology and safety	Dog: partial; Guinea pig: partial; Mouse: partial; Monkey: yes; Rat: partial	None	No
17	Trace amine associated receptor (TAAR)1 partial agonist	No	In vivo pharmacology and safety	Monkey: partial; Mouse: no; Rat: partial	None	No
18	Orexin receptor antagonist	Yes	Safety	Dog: partial; Guinea pig: partial; Rat: partial	None	No
19	P2X7 channel antagonist	No	In vivo pharmacology and safety	Rat: partial; Dog: no, Guinea pig: partial, Monkey: yes	None	No
20	Muscarinic receptor partial agonist	Yes	Safety	Dog: partial; Monkey: yes; Rat: partial	None	No
21	Nicotinic acetylcholine (nAChR) inhibitor (prodrug)	No	Safety	Dog: partial; Ferret: no; Mouse: partial; Rat: partial	Commonly used	No
22	Orexin receptor antagonist	Yes	Safety	Dog: partial; Guinea pig: partial; Mouse: partial; Rat: partial	None	No
23	Beta-glucocerebrosidase (GCase) allosteric activator	Yes	In vivo pharmacology and safety	Dog: yes; Monkey: yes; Mouse: partial; Rat: yes	Biomarker	No
24	GABA positive allosteric modulator	Yes	In vivo pharmacology and safety	Minipig: yes; Monkey: yes; Mouse: partial; Rat: partial	Symptomatology	No
25	AMPA receptor positive allosteric modulator	No	In vivo pharmacology and safety	Monkey: partial; Rat: partial	None	No

In addition to preclinical data retrieved from the IBs, the aims and results of the actual early human studies were extracted from the protocols and clinical study reports (CSRs). If available, these results were complemented with other clinical data provided in section 4 of the IB (“Effects in Humans”). For all included studies, information on the mechanism of action of the IMP and whether the clinical study was an FIH study was collected. For FIH studies, the section in the protocol describing the rationale for starting dose selection was assessed on whether the starting dose was primarily based on preclinical safety or also on in vivo pharmacological effects. As part of this, it was assessed whether the lowest preclinical and clinical pharmacologically active doses were established.

### Data analysis

Two separate analyses were performed to determine the predictability of preclinical findings in humans: one for tolerability and another for pharmacologic activity. The purpose of these analyses was to compare the tolerable and pharmacologically active ranges in laboratory animals to those found in humans. The ranges were calculated for all three exposure parameters: dose (HED), *C*_max_ and AUC.

For tolerability, the ratio between the starting level of the clinical study and the NOAEL, and the ratio between the highest well-tolerated level in the clinical study and the NOAEL were calculated. The starting level for the clinical study was defined as ‘human lower range adverse events’ (HLR_AE_), and the highest well-tolerated level in the clinical study as ‘human upper range adverse events’ (HUR_AE_).

**Fig. 1:**
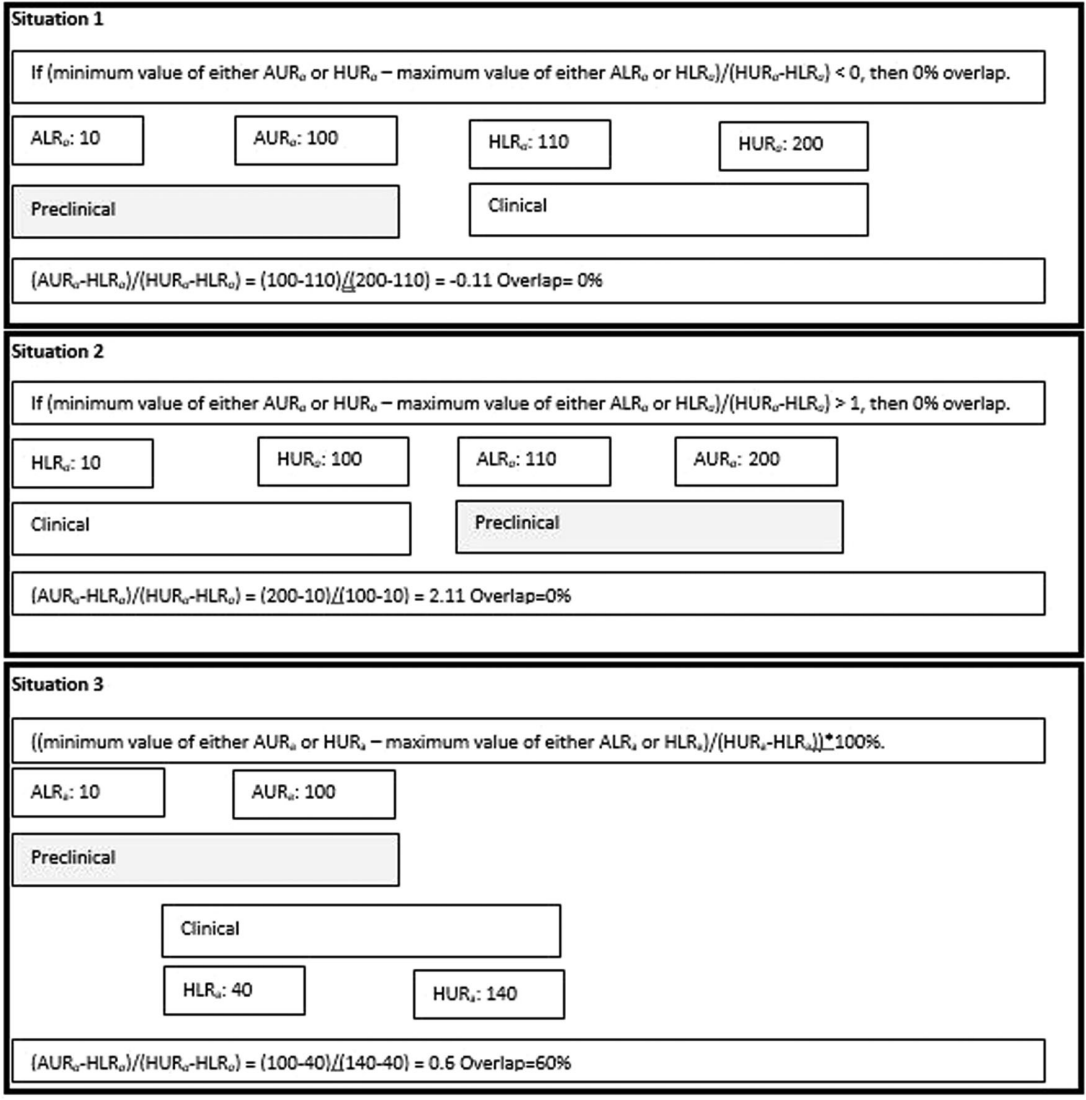
Overlap calculations. It illustrates how the calculations were performed.

The preclinical pharmacologically active range was determined by the ‘animal lowest’ and ‘animal highest’ dose-, concentration- or exposure-level, at which any effect associated with the proposed mechanism of action of the drug was reported in any animal species. These values were called ALR_*a*_ ‘animal lower range active’ and AUR_*a*_ ‘animal upper range active’. Preclinical safety findings were often observed at higher levels than the highest level tested in efficacy experiments. If the safety issues were judged to be related to a compound’s pharmacological effects, this was used to determine the AUR_*a*_. The human active ranges HLR_*a*_ and HUR_*a*_ were defined as the lowest and highest level, respectively, in which pharmacological effects associated with the proposed mechanism of action of the drug were reported. An illustration of the calculations of the overlap between the preclinical active dose range with the corresponding clinical dose ranges for each exposure parameter (HED, *C*_max_ and AUC) can be found in Fig. 1. Calculations were performed according to the formula below:

- If (minimum value of either AUR_*a*_ or HUR_*a*_–maximum value of either ALR_*a*_ or HLR_*a*_)/(HUR_*a*_-HLR_*a*_) < 0, then 0% overlap.
- If (minimum value of either AUR_*a*_ or HUR_*a*_–maximum value of either ALR_*a*_ or HLR_*a*_)/(HUR_*a*_-HLR_*a*_) > 1, then 0% overlap.
- Otherwise: ((minimum value of either AUR_*a*_ or HUR_*a*_–maximum value of either ALR_*a*_ or HLR_*a*_)/(HUR_*a*_−HLR_*a*_))*100%.

## Results

### General characteristics of studies

**Fig. 2:**
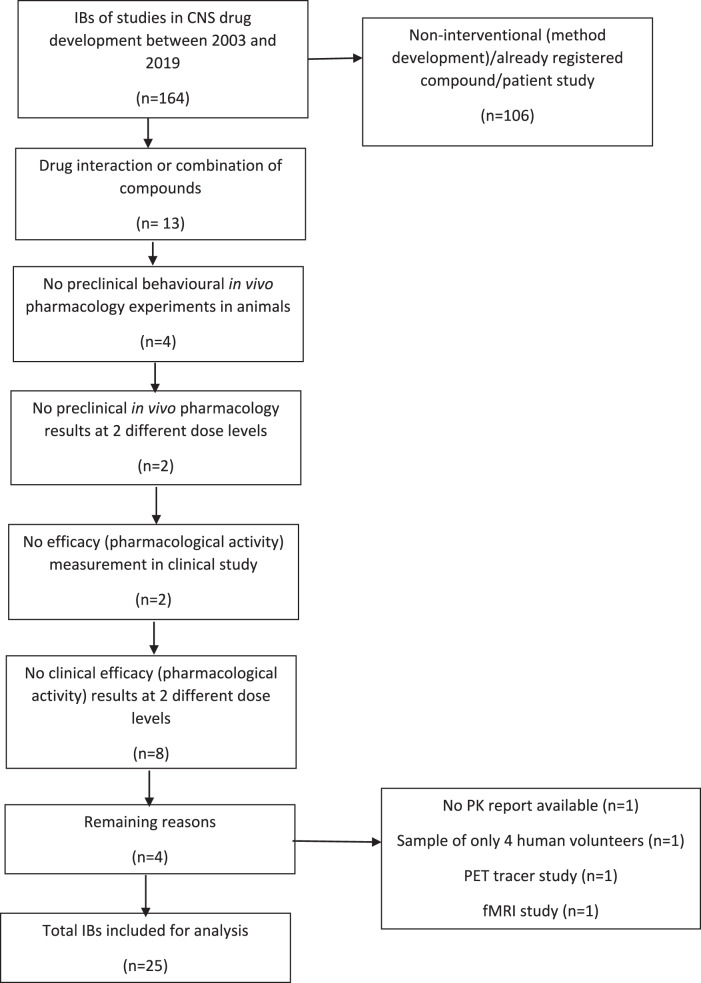
Flowchart of included studies. It depicts the reasons of why studies were excluded.

The search in CHDR’s database from 2003 up to and including 2019 returned 164 finished clinical CNS drug studies in healthy volunteers with corresponding IBs. Of these, 25 studies met the inclusion criteria (Fig. 2). The most common reasons for exclusion were studies being non-interventional (method development), or studies with a registered compound or patient studies (*n* = 106), studies focusing on drug–drug interaction (*n* = 13) and no clinical efficacy (pharmacological activity) results at two different dose levels (*n* = 8) (Fig. 2). The four studies that were excluded because there were no preclinical behavioural in vivo pharmacology experiments in animals performed concerned three studies in which only cell and cytokine responses were measured preclinically and a study in which only in vitro experiments were performed preclinically to assess the pharmacodynamics of the novel compound. The two studies that were excluded because there were no preclinical in vivo pharmacology results at two dose levels concerned one study in which several doses were tested in preclinical behavioural experiments, but only one dose had a statistically significant effect and one study in which only one dose was tested in a preclinical in vivo pharmacology experiment. These studies were not included in the analysis as the outcome could then be only 0% or 100% overlap. The two studies that were excluded because there was no efficacy (pharmacological activity) measurement in the clinical study concerned PK studies.

Of the eight studies that were excluded because there was no clinical efficacy (pharmacological activity) result at two different dose levels, there were four studies that measured a statistically significant effect in the clinical study, but only at one dose level. These studies were not included in the overlap calculations as the overlap could then only be 0% or 100%. There were four studies in which the results of the efficacy or pharmacological activity measurement in the clinical study were negative. This concerned a study measuring the effect of a single dose esketamine on driving performance compared to the effect of a placebo and positive control. A study measuring the effect of different doses of a novel compound (a selective muscarinic M 1-acetylcholine receptor agonist) on cognitive performance as measured by a battery of neurocognitive and neurophysiological tests in healthy elderly with below-average cognitive functioning. A study into the effect of different doses of a novel compound (a dual enkephalinase inhibitor) on neurocognitive and neurophysiological tasks and on a nociceptive test battery, and a study measuring CNS effects of different doses of a novel compound (guanylate cyclase stimulator) in healthy volunteers measured by a battery of neurocognitive and neurophysiological tests. In all four of these studies, the preclinical pharmacological activity experiments did demonstrate statistically significant effects.

Table 1 shows that 12 (48%) of the included studies concerned a First-in-Human (FIH) study. In five of these studies (42%), the starting dose was based on both safety and in vivo pharmacology findings. In seven (58%) studies the starting dose was only based on safety findings.

In 8 of the 25 IBs (32%) a rationale for the chosen animal model was provided. The motivations included availability (commonly used models), similar phenotype (symptoms), response to effective drugs (pharmacology), histology and biomarkers. There was no explanation as to the relevance of model choice when compared to other available options. In none of the IBs pharmacokinetic reporting was complete for all in vivo pharmacology experiments (Table 1). In most cases, PK values of behavioural or disease models in mice were not reported (Table 1). The strain and sex were frequently missing. None of the preclinical experiments in the included IBs fulfilled the criteria of internal validity as most animal experiments included a placebo arm, but blinding or randomization was not reported in any of the experiments (Table 1).

### Tolerability assessment

#### Predictions of tolerability based on human equivalent dose

Table 2 shows that in most studies (*N* = 22, 88%), the starting dose (HLR_AE_) for the clinical study was at least a (rounded) factor 10 below the HED of the NOAEL (Ratio HLR/NOAEL). This is as expected since FDA guidelines propose 10 as the default safety factor [18]. Three studies that did not apply this method were the IB2, IB4 and IB21 studies. The studies of IB2 and IB4 investigated novel cannabinoid receptor agonists. In these studies, a starting dose of, respectively, factor 1.8 and 5.3 below the HED of the NOAEL was deemed safe by the investigators, as the NOAEL was based on transient, species-specific, monitorable effects on blood pressure, and considering the safety profile of other well-known cannabinoid agonists. The study of IB21 was no FIH and the starting dose was based on the results of a previous clinical study.

**Table 2:** Safe and tolerable dose ranges.

IB number	Human equivalent dose (mg)					*C*_max_ (ng/ml)					AUC (ng*h/ml)				
	NOAEL	HLR	HUR	Ratio HLR/NOAEL	Ratio HUR/NOAEL	NOAEL	HLR	HUR	Ratio HLR/NOAEL	Ratio HUR/NOAEL	NOAEL	HLR	HUR	Ratio HLR/NOAEL	Ratio HUR/NOAEL
1	1440	5	240	288.0	6.0	7797	0	774	7797.0	10.1	113,029	0	5594	1,130,29.0	20.2
2	0.032	0.018	0.18	1.8	0.2	1	0.398	4.55	2.5	0.2	0.8	1.54	15.7	0.5	0.1
3	288	1	1000	288.0	0.3	677	0	291	677.0	2.3	2760	0	1910	2760.0	1.4
4	0.032	0.006	0.06	5.3	0.5	0.63	0	1.52	0.6	0.4	0.37	0	0.456	0.0	0.8
5	96	2.5	80	38.4	1.2	167	63	1030	2.7	0.2	1730	324	11200	5.3	0.2
6	6.14	0.5	20	12.3	0.3	49.3	0.343	73.3	143.7	0.7	274	3.1	542	88.4	0.5
7	105.6	1	40	105.6	2.6	674.25	15	497	45.0	1.4	4743	57.9	2182	81.9	2.2
8	288	5	900	57.6	0.3	780	18.9	2946	41.3	0.3	6336	261	45,286	24.3	0.1
9	97.2	10	600	9.7	0.2	2130	9.4	1430	226.6	1.5	26,800	9	5790	2977.8	4.6
10	78	0.05	0.8	1560.0	97.5	134	0	149	134.0	0.9	1763	0	417	1763.0	4.2
11	972	5	1500	194.4	0.6	4445	40.1	935.9	110.8	4.7	44,200	161.4	26,585.8	273.9	1.7
12	32.46	0.5	1.5	64.9	21.6	609	0.22	1.4	2768.2	435.0	2421	0.29	3.55	8348.3	682.0
13	58.3	0.005	0.5	11,660.0	116.6	610	0.00913	0.885	66,812.7	689.3	7584	0.245	32	30,955.1	237.0
14	58.3	0.04	100	1457.5	0.6	1230	0.229	559.3	5371.2	2.2	9850	1.6	5753	6156.3	1.7
15	1623	1	180	1623.0	9.0	42.9	0.572	98.8	75.0	0.4	108	12.1	8666	8.9	0.0
16	267.8	10	80	26.8	3.3	1270	183	1208	6.9	1.1	3570	982	3648	3.6	1.0
17	874.8	30	450	29.2	1.9	3267	66.3	1130	49.3	2.9	NR	743	14,600	NR	NR
18	973	5	200	194.6	4.9	3560	159.7	1868.9	22.3	1.9	10,900	971.3	22,906.4	11.2	0.5
19	486.9	50	450	9.7	1.1	1740	138	1180	12.6	1.5	27,200	2456	22,357	11.1	1.2
20	32.5	1	35	32.5	0.9	267	3.4	235	78.5	1.1	1990	42	2210	47.4	0.9
21	19.4	5.5	44	3.5	0.4	27.1	10.7	58.5	2.5	0.5	34.8	15.7	119	2.2	0.3
22	560	1	120	560.0	4.7	6976	97.4	4575	71.6	1.5	NR	1969	64,642	NR	NR
23	811	3	90	270.3	9.0	4433	110	3200	40.3	1.4	55,333	1600	84,700	34.6	0.7
24	1920	2.5	375	768.0	5.1	33,300	32.5	3520	1024.6	9.5	289,000	286	46,500	1010.5	6.2
25	19.44	0.3	18	64.8	1.1	359	2.93	126	122.5	2.8	1069	47.7	8882	22.4	0.1

**Fig. 3:**
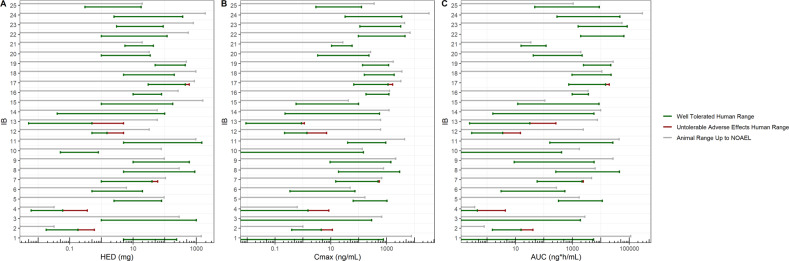
Safe and tolerable dose ranges. **A** The overlap ranges for HED are depicted. **B** The overlap ranges for *C*_max_ are depicted. **C** The overlap ranges for AUC are depicted. For both preclinical (animal) and clinical (human) studies the investigated ranges per pharmacokinetic parameter are depicted. The preclinical (animal) ranges are depicted in grey. The clinically (human) well-tolerated ranges are in green and the ranges that were well-tolerated preclinically, but not clinically are depicted in red.

For HED, the human upper tolerability range HUR_AE_ surpassed the NOAEL in 9 out of 25 studies (36%): IB2, IB3, IB4, IB6, IB8, IB9, IB11, IB14 and IB20 (Table 2, Fig. 3). In all except two of these studies, the compound was well tolerated up to the highest administered dose range (HUR_AE_), probably explaining why human doses could be escalated to levels beyond the NOAEL. Studies with IB2 and IB4 both involved experimental cannabinoid receptor agonists. The preclinical NOAEL for these compounds was based on cardiovascular side effects, which can be intensely monitored in humans and therefore be used to guide dose escalation. Although such effects also occurred in humans, they were not considered dose-limiting. For both cannabinoid receptor agonists, dose escalation was only halted when undesirable (reversible) psychiatric events occurred, which were in line with the action mechanism.

#### Predictions of tolerability based on *C*_max_

As shown in Table 2 and Fig. 3, the *C*_max_ value of the HUR_AE_ surpassed the *C*_max_ value of the NOAEL in 8 of the 25 studies (32%; IB2, IB4, IB5, IB6, IB8, IB10, IB15, and IB21). The findings with the cannabinoid receptor agonists of IB2 and IB4 were mentioned before. In the clinical studies of IB5, IB6, IB8, IB10, IB15, and IB21 the compound was well tolerated up to the highest HUR_AE_, allowing the dose levels to be escalated to levels beyond the NOAEL.

#### Predictions of tolerability based on AUC

For AUC, Table 2 and Fig. 3 show that the HUR_AE_ surpassed the AUC associated with the NOAEL in 12 out of 23 studies (52%; IB2, IB4, IB5, IB6, IB8, IB15, IB16, IB18, IB20, IB21, IB23, IB25). In all these studies (except IB2 and IB4 as explained above), the compound was tolerated well enough to be escalated to levels above the NOAEL.

#### Other observations on tolerability

In none of the clinical studies serious adverse events or irreversible adverse events, related to the investigational compound, were reported. Four human studies (IB7, IB12, IB13 and IB17) showed unacceptable adverse events at levels below values associated with the NOAEL for all exposure parameters (Table 2, Fig. 3). In the study of IB7 with a GABA receptor modulator, volunteers experienced ataxia, imbalance, tiredness and drowsiness. Symptoms of comparable nature, such as somnolence and ataxia, were also observed preclinically, albeit at higher dose levels. In the clinical study of IB12 with a histamine receptor agonist, participants reported pseudo-hallucinations and experienced hypotension. Preclinically, decreases in blood pressure were also observed, but at much higher dose levels. Pseudo-hallucinations could obviously not be observed preclinically, but behavioural changes were observed in monkeys at much higher dose levels than those given in the clinical study. In the study of IB13 with a histamine receptor antagonist, subjects experienced moderate nausea and insomnia. Preclinically, increased wakefulness was also observed, but this was considered a desired effect that was observed at similar dose levels. Emesis only occurred in dogs at much higher dose levels than in the clinical study. For IB17 with a trace amine-associated receptor (TAAR) partial agonist, cardiovascular AEs of tachycardia, palpitations and orthostatic hypotension were observed. Increased heart rate was also observed preclinically, but at much higher dose levels.

Taken together, *C*_max_ values seem to be the most accurate predictor of tolerability limits for CNS active compounds, with only 32% of clinical studies reporting well-tolerated doses above the NOAEL value, compared to 36% for HED and 52% for AUC. The percentage (16%) of clinical studies reporting unacceptable side effects at values below the NOAEL was similar for all three exposure parameters.

### Pharmacological activity assessment

#### Preclinical and clinical pharmacologically active ranges

**Fig. 4:**
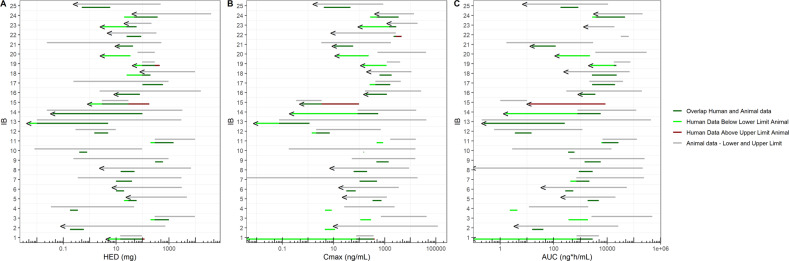
Pharmacologically active dose ranges. **A** The overlap ranges for HED are depicted. **B** The overlap ranges for *C*_max_ are depicted. **C** The overlap ranges for AUC are depicted. For both preclinical (animal) and clinical (human) studies the investigated ranges per pharmacokinetic parameter are depicted. The preclinical (animal) ranges are depicted in grey. The clinically (human) pharmacological active range that was below the range in which pharmacological activity was observed preclinically (animal) is depicted as light green. The clinically (human) pharmacological active range that was above the range in which pharmacological activity was observed preclinically (animal) is depicted as red. The overlap of preclinical and clinical pharmacological active dose ranges is depicted as dark green. < Means that the lowest dose tested already demonstrated an effect.

Eight (32%) IBs (IB2, IB5, IB6, IB8, IB18, IB22, IB23, IB24, IB25) reported statistically significant pharmacological effects at the lowest tested preclinical dose (Fig. 4). Hence, 32% of the preclinical studies did not cover the full concentration-effect range.

Nine (36%) clinical studies (IB1, IB13, IB14, IB15, IB16, IB19, IB20, IB21, IB23) showed a pharmacological effect at all administered doses (Fig. 4). For one study (IB23 with a GCase modulator) both the preclinical and clinical lowest tested dose was effective, meaning that no no-effect level was defined.

#### Predictions of pharmacological activity based on HED

On average, HED was the best predictor of pharmacologically active ranges, with 84% overlap between preclinical and clinical pharmacologically active ranges (Table 3, Fig. 4). For one compound (IB20) there was no overlap between the preclinical and clinical ranges for HED or any other exposure parameter. This involved a muscarinic receptor partial agonist. Memory testing showed improvement in healthy volunteers, at lower levels than in the preclinical experiments. Memory functioning in the clinical study was tested using the NeuroCart, which consists of a battery of drug-sensitive neurophysiological and cognitive tests [19]. Possibly the NeuroCart is more sensitive to drug effects than preclinical models available to test memory functioning, explaining why drug effects in humans were observed at lower exposure levels than in animals. Further dose escalation in the clinical study was prevented by adverse events that could be expected with muscarinergic agonists, such as increased blood pressure and hypersalivation. Blood pressure increases were also described in the IB in rats, at a similar HED but with higher *C*_max_ and AUC values.

**Table 3:** Pharmacologically active dose ranges.

IB	Human equivalent dose (mg)					*C*_max_ (ng/ml)					AUC (ng*h/ml)				
	ALR	AUR	HLR	HUR	% Overlap	ALR	AUR	HLR	HUR	% Overlap	ALR	AUR	HLR	HUR	% Overlap
1	14.4	96	5	120	71	76.6	367	0	403	72	719	4931	0	2870	75
2	0.096	736	0.18	0.6	100	11.9	118,732	4.55	11.5	0	4	26,232	15.7	41.4	100
3	288	9600	200	1000	89	707	43,324.6	111	291	0	2590	492,674	362	1910	0
4	0.034	48	0.18	0.36	100	26	2424	4.66	8.51	0	12	1968	2.29	4.4	0
5	28.8	4800	20	60	78	27	1210	334	749	100	225	20,700	1860	4870	100
6	8.54	3072	10	20	100	19.4	3530	31.5	73.3	100	40.87	54,900	272	542	100
7	0.355	3000	10	40	100	0	19,305	104	497	100	707	238,848	423	2182	84
8	2.88	6912	15	50	100	8.7	8990	61	205	100	0	216,000	875	2895	100
9	0.243	972	300	600	100	51.9	15,619	477	1430	100	396.5	250,938	1440	5790	100
10	0.008	97.2	0.4	0.8	100	0.177	15,855	149.3	156	100	2.82	14,523	347	606	100
11	291.6	9738	200	1500	93	1660	16,450	479.3	868.9	0	6741	130,500	6113.1	26,585.8	97
12	0.29	9.72	1.5	5	100	2.1	700	1.4	7.1	88	0.6	1189	3.55	14.97	100
13	0.010	2961	0.005	5	100	0.074	42,435	0.009	1.144	94	0.205	435,485	0.245	262.66	100
14	0.024	3246	0.04	100	100	87.2	17,000	0.229	559.3	84	775	122,000	1.6	5753	87
15	2.9	29.16	1	180	15	0.342	3.42	0.572	98.8	3	1.014	10.14	12.1	8666	0
16	2.4	16230	10	80	100	169.8	26,371	183	1208	100	297	200,000	982	3648	100
17	0.243	972	100	600	100	429.7	4200	261	1660	88	2180	39,007	2750	19,800	100
18	97.2	9720	25	200	59	239	11,113	631.58	1868.9	100	290	69,700	2703.2	22,906.4	100
19	97.2	291.6	50	450	49	1214	4013	138	1180	0	17909	74,330	2456	22,357	22
20	64.9	291.6	3	35	0	523	41,300	13.7	235	0	3620	301,000	134	2291	0
21	0.024	519.4	11	44	100	3.3	1740	10.7	58.5	100	1.7	2990	15.7	119	100
22	5.6	336	25	90	100	9.6	2698	2230	4575	20	NR	NR	32,831	64,642	100
23	24.3	218.7	3	60	63	1484	19,286	110	2820	49	NR	NR	1600	18700	100
24	48.6	39,600	20	375	92	496	14,000	271	3520	93	3780	212,000	2790	46,500	98
25	0.29	486	0.5	6	100	1.9	883	4.19	45.99	100	8.28	10800	181	839	100
Average					84					64					78

#### Predictions of pharmacological activity based on *C*_max_

The preclinical and clinical pharmacologically active range showed overlapping *C*_max_ values in 64% of studies. There was no overlap for IB2, IB3, IB4, IB11, IB19 and IB20 (Table 3, Fig. 4).

In the clinical studies of IB2 and IB4, some participants experienced typical mental effects of cannabinoid receptor agonists, at *C*_max_ values well below preclinically active levels. The psychiatric effects were considered dose-limiting in view of the anticipated therapeutic indications (analgesia and sedation).

In the clinical studies of IB3 and IB11, both with an orexin antagonist, preclinical pharmacological effects were observed at higher *C*_max_ levels than in the clinical study. It is possible that more sensitive PD measurements for orexin antagonists are available in humans (using the NeuroCart) than in animals, to detect (subjective) reduced alertness, attention and vigilance.

In the clinical study of IB19 with a subtype selective purine antagonist, decreased interleukine-1β release was observed in humans at a somewhat lower *C*_max_ value than preclinically, suggesting that humans are more sensitive to the effects of purine antagonists than animals.

Pharmacological activity was measured at a lower *C*_max_ value in humans than in animals in the clinical study of IB20 with a muscarinic receptor partial agonist, again suggesting that memory tests are more sensitive to this class of compounds in humans than in animals.

#### Predictions of pharmacological activity based on AUC

Pharmacologically active AUC ranges in animals and humans overlapped in 78% of studies. There was no overlap between animals and humans for AUCs of compounds in IB3, IB4, IB15 and IB20 (Table 3, Fig. 4). In the clinical study of IB3 with an orexin antagonist, the preclinical effects were observed at higher AUC levels than in the clinical study. It is possible that—partly subjective—PD measurements are more sensitive to orexin antagonists in humans than in animals [19]. However, the overlap between the preclinical and clinical pharmacologically active AUC range in IB11 with an orexin antagonist as well was 97%. In general, the PK profile of this orexin antagonist was much more comparable between animals and humans than for the orexin antagonist of IB3. The same was found for the two cannabinoid receptor agonists. The AUC overlap of pharmacologically active ranges for the compound of IB2 was 100%. For IB4 there was no overlap because, in the clinical study, dosing was stopped for unacceptable (albeit pharmacological) mental effects at lower AUC levels causing detectable pharmacological effects in animals.

In the clinical study of IB15 with a subtype selective nicotinic receptor agonist, the preclinical pharmacologically active range was lower than the clinical active range. However, the lowest dose in humans already showed pharmacological activity, meaning that there could be an overlap, but this was not assessed. For the muscarinic receptor partial agonist of IB20, the NeuroCart could demonstrate pharmacological activity in humans at lower AUCs, than where effects occurred in animals.

Overall, the preclinical data predicted the pharmacologically active range in humans to a high degree, as indicated by an overlap of ≥ 80% in 18 out of 25 (72%) for HED, 15 out of 25 (60%) for *C*_max_ and 19 out of 23 (83%) for AUC. A particularly poor preclinical prediction of the clinical active range (as indicated by ≤ 20% overlap) was shown in 2 out of 25 studies (8%) for HED, 7 of 25 (28%) for *C*_max_ and 4 out of 23 studies (17%) for AUC.

### Therapeutic efficacy assessment

Therapeutic efficacy studies were reported for only six of the 25 included compounds in our paper. For three out of the six compounds, there was a large overlap between the therapeutic effective ranges in patients and the preclinical and human pharmacologically active ranges. For the three other compounds, the therapeutic effective ranges were lower than the preclinical pharmacologically active range, but they corresponded closely with the clinical pharmacologically active ranges in healthy volunteers.

## Discussion

The results show that, in general, tolerable dose ranges for clinical studies with novel CNS active compounds can be reasonably well predicted from preclinical data. Overall, *C*_max_ corresponding to the preclinical NOAEL was the best predictor of the tolerable range in humans, although the observed adverse effects in animals (or any other dose-limiting effect) did not occur in 32% of the healthy volunteer studies. HED and AUC predictions based on this ‘default’ safety level were even more conservative (with poor predictability in 36% and 52%), particularly when the effects could be readily monitored in healthy subjects and used for dose escalation (e.g., using intensive cardiovascular monitoring, or repeated NeuroCart measurements for CNS-effects).

In 4 out of 25 studies (24%), the highest tolerated (administered) doses in humans (HUR_AE_) were much lower than expected based on the NOAEL for all three exposure parameters. This concerned the clinical study of IB7 (GABA modulator), IB12 (histamine agonist), IB13 (histamine antagonist) and IB17 (TAAR partial agonist). In three cases (IB7, IB13 and IB17) the dose-limiting AEs reported by volunteers, including ataxia, hypotension, drowsiness, insomnia and nausea, were observed preclinically as well, but only at higher dose levels. Thus, a considerable proportion of CNS active agents (28%) seem to have more prominent effects in humans than animals. For some compounds, dose escalation was limited by psychiatric side effects, which are difficult to observe preclinically. This was the case for the histamine receptor agonist of IB12 at doses well below NOAEL. Mental effects also limited the dosing of the two cannabinoid receptor agonists (IB2 and IB4), but here the highest tolerated dose in humans (HUR_AE_), was higher than the values associated with the NOAEL that was based on cardiovascular effects. These results emphasize that in FIH studies with CNS active compounds, researchers should pay special attention to the psychiatric effects of new compounds, as these cannot be reliably predicted from animal experiments.

In all studies included in this report, the observed adverse events were exaggerated pharmacological effects in line with the working mechanism of the compound and therefore predictable based on preclinical data. This illustrates the importance of monitoring pharmacological effects of compounds based on the mechanism of action. Monitoring based on translatable and thus predictable pharmacological mechanisms of actions can also include important off-target effects, which in the IB are presented as ex vivo or in vitro pharmacological binding studies.

The average overlap values of preclinical and clinical pharmacologically active dose ranges demonstrate that the prediction of clinical pharmacologically active dose ranges based on preclinical data of behavioural experiments is fairly reliable. With an average overlap of 84%, the HED was the best predictor for the pharmacologically active dose range. Possibly, this reflects a bias in reporting as the MABEL or PAD were most often based on the HED in the IBs included in this report. When looking at the PK parameter with the highest percentage of high preclinical and clinical active dose range overlap, AUC was the best predictor with 83% of the compounds having more than 80% overlap.

In cases where no overlap between preclinical and clinical pharmacological active dose ranges could be observed, humans were more sensitive to the effects of the compound. In the clinical studies with cannabinoid receptor agonists, it was not possible to dose up to the levels of desired pharmacological (analgesic, sedative) effects due to unacceptable psychiatric (but still pharmacological) effects observed at lower dose levels. Not only for psychomimetic effects but also for some other CNS effects, more sensitive methods are available in humans than in animals. Complex measures of memory or eye-hand coordination in the NeuroCart showed effects of orexin antagonists and cholinergic/muscarinergic agonists, at lower levels in humans than predicted from animal models.

Four studies were excluded from the quantitative analysis of overlapping exposure ranges because no statistically significant effects were observed on measurements of pharmacological activity/pharmacodynamics in the clinical study. In all four of these studies, statistically significant effects were observed in preclinical, behavioural experiments, but not in humans. One of the omitted studies in humans was not designed to measure pharmacodynamic effects of the compound, but to assess continuous driving performance (after *t*_max_), which explains why no effect was observed. The other three studies concerned compounds that aim to modulate neuronal processes in the brain in the longer term instead of in the acute phase, which might explain why no statistically significant effect was observed in the single-dose clinical studies.

The EMA guideline on how to determine the starting dose for an FIH study was updated after the TGN1412 study. In this new guideline, published in 2007, it was recommended to base the starting dose not only on the NOAEL but also include the MABEL [8]. In our sample of FIH studies performed between 2003 and 2019, in 58% of the included studies, the starting dose for the clinical study was based on preclinical safety experiments (NOAEL) only. One of these studies was performed prior to 2007. This percentage is in line with other publications reporting that the NOAEL-based approach is still the most common method to determine the starting dose for an FIH study [11–13].

In line with previous research, our data show that important details of animal studies are poorly reported in IBs [17]. In none of the IBs blinding or randomization of the preclinical experiments was reported and most lacked important information, such as animal sex or route of administration. PK measurements were often missing for animal models of behaviour or disease. Although regulatory guidelines do not require the reporting of PK-analyses in each preclinical study, the translation to effective human dose ranges is not possible without exposure data. Since poor reporting of study design is often associated with an overestimation of efficacy outcomes, it means that ethics committees and other regulatory bodies could be allowing first-in-human trials to start on the basis of spurious results [17, 20].

For only six of the 25 compounds therapeutic efficacy study results were reported. For three of those, the therapeutic effective ranges in patients were lower than the preclinically pharmacologically active range, but in all cases, there was a good overlap between the pharmacological effect ranges in healthy volunteers and the therapeutic dose range. This relatively high translatability is contradictory to existing literature reporting high failure rates of translation of new investigational compounds that seem effective in preclinical experiments but fail in clinical therapeutic studies [21]. These findings may be biased to some extent as the decisions to advance these compounds to clinical trials in patients relied on consistent results from the preclinical and human phase I studies. Next to that, phase I studies often solely focus on tolerability, safety and pharmacokinetics instead of also including relatively basic human pharmacological characteristics of new compounds such as blood-brain barrier penetration, as done in included studies [22]. Another factor often cited as a cause for the high attrition rate in CNS drug development is the limited knowledge of receptor occupancy [23]. A possible solution to this problem is to perform more PET studies to study receptor occupancy [23]. The high attrition rate in CNS drug development can also be explained by poorly understood human diseases as psychiatric disorders are usually diagnosed based on a cluster of symptoms instead of a biological basis [24]. This leads to several problems, such as the animal model being a mismatch or simplification of the human disease. There are current initiatives to overcome these problems, such as the Research Domain Criteria (RDoC) initiative introduced in 2009, which aims to more precisely link treatment targets to dysfunctional mechanisms relevant to clinical manifestations [24]. By doing so, biomarkers aiming to characterize the pharmacological activity of novel compounds in early-phase clinical trials are being developed as recommended in several publications [19, 22, 25].

While the trends identified in our study are worth investigating, the limited number, diversity, and non-randomness (only studies in our own research institute were included) of the included studies make our findings suggestive rather than confirmatory. We performed this overview solely with IBs of drugs for which at least two (pharmacodynamically) active doses were identified in phase I trials to allow a comparison between animal and human ranges. The lack of a dose range meant that we excluded six studies because preclinical efficacy was only established at one dose level or there was no preclinical in vivo efficacy data and ten studies because clinical pharmacodynamics was only established at one dose level or there was no clinical pharmacodynamic data (Fig. 2). As such, these data cannot be used to compare the HED, *C*_max_ and AUC regarding their ability to predict the presence of an effect in humans. Next to that, we used linear inter- and extrapolation to determine missing pharmacokinetic parameters in animals. Although such a strategy is common practice, it might lead to prediction inaccuracies for drugs with a non-linear pharmacokinetic profile, and PK/PD-based analyses might have been more reliable (albeit unfeasible owing to the lack of data in many cases). Also, the analysis was limited to studies of unregistered compounds mostly in healthy volunteers. Despite these limitations, our sample is likely representative of IBs in practice. IBs are all investigators have at their disposal when they study the pharmacokinetics, pharmacodynamics and tolerability of a new CNS active compound.

In this report, we applied the IB-derisk tool on a selection of 25 IBs and compared the predictions of tolerable and pharmacologically active dose ranges based on preclinical data to the results of clinical studies. The results demonstrate that tolerable and pharmacologically active dose ranges in clinical studies can be reasonably well predicted from preclinical data. Tolerability was best predicted by *C*_max_ and pharmacologically active ranges by HED or AUC. We noted that despite recommendations by the EMA to base the starting dose on both NOAEL and MABEL, the starting dose is often solely based on the NOAEL. In line with current literature [17], the internal validity of preclinical experiments was poor and preclinical in vivo CNS experiments are often performed without reporting PK results. The translation of preclinical to clinical studies would benefit from complete and comparable reporting of PK measurements of both toxicity and efficacy experiments. This report further demonstrates that an integrated presentation of the contents of the IB, such as provided by the IB-derisk tool, can improve the translatability of preclinical to clinical data [10, 14, 16].

Box 1 ▓

- *Pharmacokinetics (PK)*: PK covers the processes of "what the body does to the drug". More specifically, PK usually describes in a quantitative way how much drug is in the body. It includes absorption, distribution, metabolism and excretion [26].
- *Pharmacodynamics (PD)*: Pharmacodynamics describes what the drug does to the body. It is a detailed study of how drugs act and tries to answer the question whether a drug provides a meaningful pharmacological action [26].
- *Investigational Medicinal Product (IMP)*: “a pharmaceutical form of an active substance or placebo being tested or used as a reference in a clinical trial, including products already with a marketing authorization but used or assembled (formulated or packaged) in a way different from the authorised form, or when used for an unauthorised indication, or when used to gain further information about the authorised form” [27].
- *Human equivalent dose* (HED): Dose in humans equivalent to specific species, conversion factors are provided by the FDA [26].
- Maximum concentration (*C*_max_): Maximum or peak concentration of a drug observed after administration [26].
- *Area under the curve* (AUC): The area under the plot of plasma concentration of a drug versus time after dose. AUC values provide insight into the total exposure to a drug and its clearance rate from the body [26].
- No observed adverse effect level (NOAEL): The maximum dose in animal species that does not produce a significant increase in adverse events when compared to those in the control group [28].
- Minimal anticipated biological effect level (MABEL): Exposure level in humans at which biological effect is anticipated. The MABEL is based on PD effects in non-clinical studies. To establish the MABEL, receptor occupancy and target binding studies comparing animal and human cell lines should be taken into account [28].
- Pharmacologically active dose (PAD): The lowest dose tested in animal species with intended pharmacological activity [28].
- Anticipated therapeutic dose (ATD): Dose range at which an exposure level leading to therapeutic efficacy is expected [28].
